# Management of Ear Keloids Using Surgical Excision Combined with Postoperative Steroid Injections

**DOI:** 10.29252/wjps.8.3.338

**Published:** 2019-09

**Authors:** Ali Akbar Mohammadi, Sina Kardeh, Gholam Reza Motazedian, Soheil Soheil

**Affiliations:** 1 Shiraz Burn and Wound Healing Research Center, Division of Plastic and Reconstructive Surgery, Department of Surgery, Shiraz University of Medical Sciences, Shiraz, Iran;; 2 Student Research Committee, Shiraz University of Medical Sciences, Shiraz, Iran;; 3 Cell and Molecular Medicine Student Research Group, Shiraz School of Medicine, Shiraz, Iran;; 4 Faculty of Medicine, Tehran University of Medical Sciences, Tehran, Iran

**Keywords:** Ear, Keloid, Piercing, Excision, Steroid, Triamcinolone acetonide

## Abstract

**BACKGROUND:**

Ear keloids are a challenging problem that affect people of different races with substantial aesthetic consequences. Various types of adjuvant therapies, including intralesional corticosteroid injection are advocated to lower recurrence following excision. We investigated the efficacy of a protocol combined of excision and postoperative intralesional triamcinolone acetonide (TA) injection for treating earlobe keloids in a group of Iranian female patients.

**METHODS:**

A retrospective analysis of 21 patients representing 31 ear keloids treated by a single physician between 2013 and 2017 was conducted. All keloids occurred after ear piercing in female cases. Postoperative intralesional TA injection was administered once monthly and continued for several months based on the patients’ clinical progress. Results were assessed according to Kyoto scar scale.

**RESULTS:**

The patients’ mean age was 24.29 years and ranged from 16 to 40 years. After the surgery, the follow-up period ranged from 10 to 29 months (mean: 15.93 months) and patients were given TA intralesional injections 3 to 6 times (mean: 4.22 times) with no complication or adverse effect. Of the treated keloids, success was achieved in all of 31 keloids (100%) and final evaluation revealed that the mean Kyoto scar scale was significantly decreased. No recurrence occurred.

**CONCLUSION:**

Surgical excision followed by postoperative intralesional TA injection can be suggested as the primary protocol for the treatment of ear keloids considering its durable results and economic advantage.

## INTRODUCTION

Keloids are the result of pathological wound healing in which the raised scar tissue extends beyond the original margins with rare spontaneous regression. The normal interaction of numerous genes and molecules leads to the 4 overlapping phases of cutaneous healing including coagulation and hemostasis, inflammation, proliferation and finally remolding.^1^ Prolonged inflammatory phase results in histopathological imbalance seen in keloid formation characterized by excessive deposition of dermal connective tissue. This phenomenon is a result of increased collagen synthesis in the fibroblasts and decreased collagen decomposition after dermal or subcutaneous tissue injury.^2^

Although keloids may appear with unrecognized origin, this process commonly occurs in susceptible individuals after superficial or deep cutaneous trauma such as operations, ear piercing, trauma, burns and some cutaneous disorders.^3^ Risk factors include younger age, pigmented skin, mobile sites with high tension and genetic predisposition.^4^ Any traumatized region of the body including abdomen, chest, shoulder, and upper back can be a potential site for keloid appearance. However, the head and neck region, especially earlobes and the helix of the auricle are the most common sites for keloid formation.^5^

Since these lesions are conspicuous, management of the unfavorable cosmetic disfigurement and psychological problems is challenging for both surgeons and patients.^6^ In addition, further morbidities related to keloids such as pain, pruritus, ulceration, and restriction of motion are common concerns that can be of substantial burden to the mostly young patients of this condition, especially if they occur in the facial region.^7^ Considering that keloids are often resistant to treatment and a relapsing course is frequently observed in ear keloids, deploying multiple modalities has been proved to profoundly enhance the efficacy of managing these conditions.^7^

Among the numerous therapeutic options, combining surgical interventions with radiation, pressure therapy, cryotherapy, silicone-gel sheeting, laser treatment, antitumor or immunosuppressive agents and intralesional corticosteroids have been highly advocated.^8^^-^^13^ In this study, we tried to outline the results of our experience in treating 21 patients with earlobe keloids utilizing a combination of surgical excision and post-operative intralesional corticosteroid injection as an adjuvant and economical treatment in an Iranian population.

## MATERIAL AND METHODS

In this prospective interventional study, 21 female patients who developed keloid after earlobe piercing were consecutively enrolled for surgical excision followed by intralesional triamcinolone acetonide (TA) injection. Convenience sampling was used to select participants among patients referring to our plastic surgery clinic between December 2013 and March 2017. Before recruiting patients in the study, current available treatments and the advantages and risks of the proposed method comparing to other alternatives in achieving the expected satisfactory results were thoroughly explained. Level of evidence was IV and it was a therapeutic study.

**Fig. 1:**
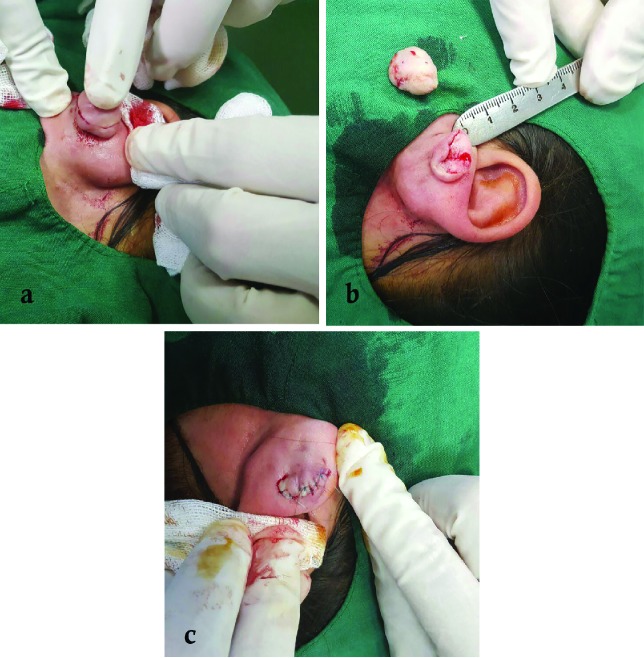
Intraleisonal excision of keloid when closure of excision site is not possible with complete excision of keloid. Incision is made leaving a remnant of keloid tissue (a). Excision of keloid except some remnant of its surrounding superficial parts (b). Using keloid superficial part as local flap for closure of wound without tension (c).

Complete removal of the fibrous core of keloid was conducted under local anesthesia using 1% lidocaine and 1:100,000 epinephrine and making incision within the edge of keloid, while raising the overlaying marginal skin. In order to maximize cosmetic outcomes, efforts were made to minimize tension on closure. So based on our judgement, if closure of keloid excision site was estimated to be possible without tension, extralesional excision was planned. However, if closure of excision site was not possible without tension, intralesional excision was performed (Figure 1).

Following the primary closure of wounds with one layer of 6-0 nylon sutures, a simple dressing consisting of sterile gauze and topical tetracycline antibiotic ointment were applied for 48 hours. Clinical evaluations were made 1 week post operation and then in a monthly follow-up program. Scar improvement and also development of any complications were re-examined by the surgeon in every visit. At first visit after the surgery and also in case of scar elevation or hardness in any of the following visits, a total of 4-10 mg intralesional injection of TA from a 20 mg/cc solution (diluted in 2% lidocaine solution) was administered once per month for several months based on the size and clinical progression of the remaining lesions. 

Kyoto Scar Scale was employed to evaluate results from both objective and subjective standpoints. The objective signs are redness, hardness, and elevation graded into 3 levels (0-2). On the other hand, subjective symptoms include itching and pain which are graded into 2 levels (0-1).^13^ Finally, adding the points together determined the total scores with four overall categories (Table 1). An informed written consent was obtained from each participant of this study. Besides, the study procedures were approved by the Ethics Committee of Shiraz University of Medical Sciences. Data collection was conducted within the study period and then transported to SPSS software (Version 20, Chicago, IL, USA) for analysis. Descriptive data is presented as mean±SD. Statistical analysis was done by using Wilcoxon Signed Ranks Test. A P value less than 0.05 was considered statistically significant.

**Table 1:** Keloid scoring according to Kyoto scale

Evaluation	Score
Excellent	0
Good	1-2
Fair	3
Poor	4-8

## RESULTS

**Fig. 2:**
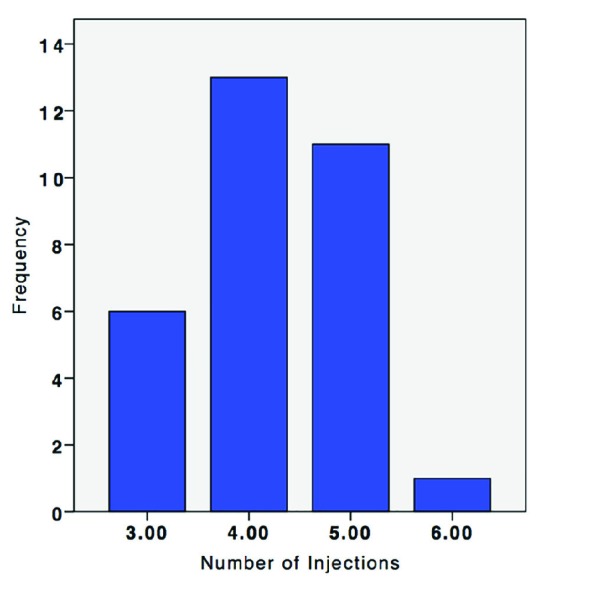
Frequency of TA injections after surgical excision

We evaluated a total of 21 female patients with the mean age of 24.29 (Min=16, Max=40, SD=6.64) year. Eleven patients had unilateral and 10 patients had bilateral earlobe keloid after ear piercing. Intralesional excision was performed on 22 (71%) of keloids, while 9 (29%) were managed by extra-lesional excision. None of the patients were lost to follow-up. Postoperative follow-up duration ranged from 10 to 29 months with the average of 15.93 months (SD=5.79). A minimum of 3 postoperative intralesional corticosteroid injections was done. The maximum number of administered injections was 6, which was only the case in one lesion (Mean=4.22) (Figure 2). 

**Fig. 3:**
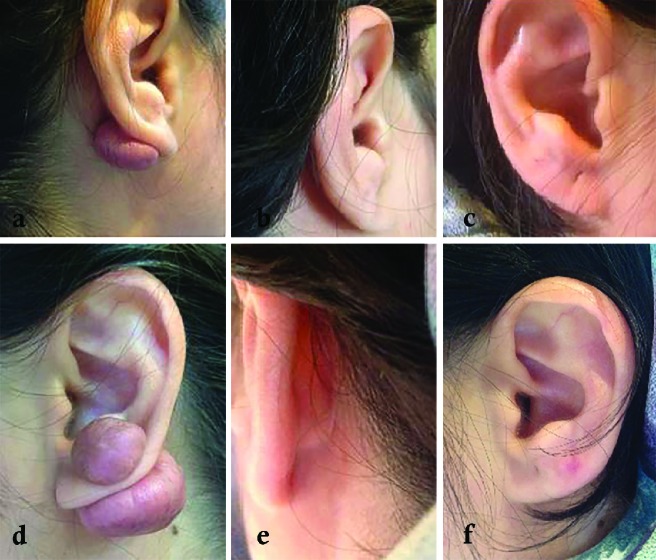
Right side ear keloid in the patient before intervention (a), after completion of treatment (b, c). Left side ear keloid in the same patient before intervention (d), after completion of treatment (e, f).

**Fig. 4:**
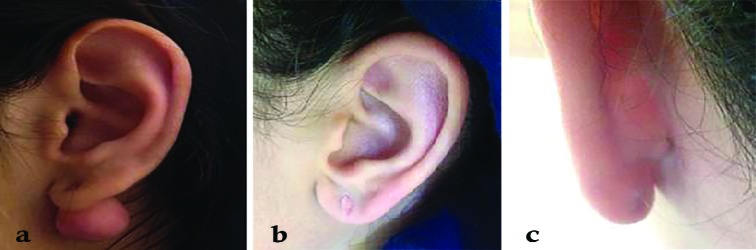
Left side earlobe keloid (a), 13 months after treatment (b, c)

**Fig. 5:**
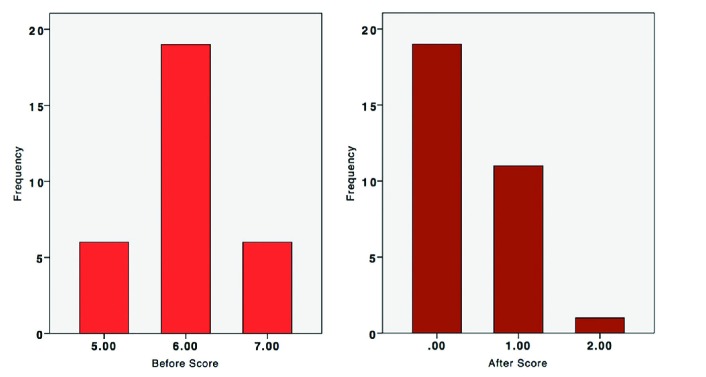
Kyoto Scar Scale before and after intervention

None of the lesions showed any sign of keloid recurrence. Excellent treatment results were achieved in 61.3% of lesions (n=19, Kyoto score=0) (Figures 3, 4) and 38.7% of showed highly acceptable outcomes (n=11, Kyoto score=1, n=1, Kyoto score=2). The mean Kyoto scar scale score was 6.00 (Min=5, Max=7) before intervention, which decreased significantly to 0.41 (Min=0, Max=2) after treatment (Wilcoxon Signed Ranks Test, P<0.005) (Figure 5). 

## DISCUSSION

In spite of a considerable amount of information now available in the literature about histomorphological structure of keloids, optimal treatment to patients is still a challenge.^14^ There is no guideline that can define the efficacy in the wide variety of treatment techniques as represented by a low recurrence rate and satisfactory aesthetic result for management of keloids.^15^ Based on the particular localization, various nonsurgical therapeutic procedures are used by surgeons as a combination with surgical excision.^15^

The reported success rate varies markedly with recurrences range from 50 to 100% in case of excision alone to lower recurrence rates when different therapeutic methods are implemented together.^14^^,^^16^ Our experience showed that surgical excision and postoperative intralesional injection of TA is an efficacious and well-tolerated therapeutic modality for earlobe keloid management. Delicate surgical excision, closure of the wound without tension and strict adherence to follow-up regimen are pivotal in the success rate of this treatment option. Early follow-up is mandatory to decrease postoperative risk of recurrence.^17^

So far, there is no complete understanding of the underplaying pathomechanism of keloid formation. Besides, lack of a reliable animal model has hindered research efforts for detection of the exact molecular pathways in human skin.^18^ However, it is known that certain individuals have higher susceptibility to be predisposed to this process in presence of a trigger, such as different types of skin trauma.^19^^,^^20^ Corticosteroids act initially by suppression of inflammatory process in the wound site and secondarily by impairing fibroblast growth and inhibiting a2-macroglobulin, thereby increasing collagen degradation and repressing glycosaminoglycan synthesis. Moreover, TA injection can inhibit vascular endothelial growth factor and transforming growth factor (TGF)-β1 and induce scar regression.^21^^-^^23^

Corticosteroid injections, mainly TA, have been a major role player in the treatment for pathological scars since the mid-1960s. Intralesional TA injection is effective in flattening the keloid lesions in the majority of patients, with response rates from 50 to 100 percent.^24^ Since postoperative TA injection effectively prevents recurrence of keloids following surgical excision, it is considered as an excellent therapy for recalcitrant lesions that have failed previous modalities.^25^^,^^26^

Enhanced clinical outcomes have been reported when combining TA with surgical excision which is better than using either one as monotherapy. Beside softening the keloid, TA is known to reduce symptoms, such as itching sensations and tenderness. Serial use of TA given in one to three injections per month and in concentrations ranging between 10 and 40 mg per ml can be continued until an acceptable therapeutic or cosmetic result is achieved and before the side effects become problematic.^27^^,^^28^

Major side effects include thinning and atrophy of the skin and subcutaneous tissues, telangiectasia, hypopigmentation and further necrosis of the local skin. These complications can be minimized by adjusting the dosage and correct selection of injection depth in the mid-dermis. Pain is also a common side effect and can be considered as the main disadvantage of TA injection.^28^ Given this, it is critically important to prevent treatment abandonment by the patients via attenuating injection associated pain with the addition of lidocaine. Moreover, several advantages of TA such as cost-effectiveness, and no need for specific equipment can offset the drawbacks to its use.^29^

There are several studies that have employed a combination of surgical excision and corticosteroid injections for treatment of ear keloids. However, in most of these studies multimodal approaches are used and different other adjuvant therapies mainly pressure splints were also applied.^29^^-^^31^ Similar to our investigation, in a study of Korean female patients with mean age of 24 years (ranged from 15 to 32 years), Jung *et al.* evaluated the efficacy of excision combined with pre and post-operative intralesional TA injection for treating eighteen earlobe keloids occurred after ear piercing.^32^

After the surgery, TA injections were continued once per month from 2 to 13 times depending on the patient’s clinical progress with mean follow-up period of 18.5 months (ranged from 4 to 42 months). Of the treated keloids, only three recurred (16.6%) and eleven showed good results (61.1%). Authors reported no complications from the TA intralesional injection.^32^ Furthermore, in a systematic review and meta-analysis study by Shin *et al.*, the authors compared the effectiveness and recurrence rates of TA and radiation therapy as postoperative adjuvant treatment modalities for ear keloids after surgical excision. After identifying twenty five studies which were published before August of 2015, they concluded that both TA and radiation therapy can provide excellent results for ear keloids after surgical excision without a significant difference between the two treatments.^33^

In summary, during the 34 months follow up of this study, TA injection soon after surgery caused a significant decrease in the Kyoto scar score with no recurrence or post intervention complications. Safety and cosmetic results of the method used in this study were acceptable and similar to what mentioned in many previous surveys. While other treatment options have not yet proven their superior efficacy, the results of this series might represent a better view of efficient treatment in patients with ear keloid. Although a 100% success rate is comparable if not better than any reported literature, further surveys with more patients should be performed to confirm the efficacy of a universally accepted method in large populations.

## CONFLICT OF INTEREST

The authors declare no conflict of interest.
